# Cross-Sectional Analyses to Assess the Clinical Safety and Effectiveness of Bisoprolol in Patients With Non-obstructive Coronary Artery Disease Who Underwent Percutaneous Coronary Intervention: A Post-hoc Analysis

**DOI:** 10.7759/cureus.75021

**Published:** 2024-12-03

**Authors:** Brian Pinto, Girish R Kulkarni, Soumitra Kumar, Arup Deb, Louie Fischer, Amit Khandelwal, Krishnaprasad R Korukonda, Rathish Nair

**Affiliations:** 1 Cardiology, Swarna, Mumbai, IND; 2 Medical Affairs, Torrent Pharmaceuticals Ltd., Ahmedabad, IND; 3 Cardiology, Cardiac Consultation Clinic, Kolkata, IND; 4 Cardiology, Cardio Care, Agartala, IND; 5 Cardiology, Malankara Orthodox Syrian Church Medical College, Kolenchery, Ernakulam, IND; 6 Cardiology, City Heart Cardiac Centre, Kota, IND; 7 Medical Strategic Affairs, Torrent Pharmaceuticals Ltd., Ahmedabad, IND

**Keywords:** bisoprolol, blood pressure, heart rate, hypertension, left ventricular ejection fraction, nonobstructive coronary artery disease

## Abstract

Introduction: Elevated central aortic pressure, cardiac output and peripheral vascular resistance contribute to high morbidity in relation to end organ dysfunction in obstructive and non-obstructive coronary artery disease (NOCAD) cases despite revascularization. Bisoprolol preempts further progression of left ventricular dysfunction in such cases due to anti-ischemic and anti-hypertensive effects, further extending its evaluation in local Indian settings.

Methods: Post-hoc analyses of NOCAD patients with epicardial stenosis (N=378, 30 to 70% stenosis) from cross-sectional analyses conducted across eighty centers in India. Local ethics approval for study documents and endpoints for analyses was conducted in adherence to ICH-Good Clinical Practice (GCP) and Declaration of Helsinki guidelines. Descriptive and analytical statistics were performed using SPSS Version 29.0.1.0 (IBM Corp., Armonk, NY, USA).

Results: Per-protocol analyses of NOCAD (N=378) showed (mean) age: 58.63 years (286 males and 92 females); mean weight: 75.49kg; mean BMI: 27.78kg/m^2^ and baseline left ventricular ejection fraction (LVEF): (46.85%). Prevalent risk factors include hypertension (100%), dyslipidemia (51.85%), smoking (24.07%), type 2 diabetes (59.52%), stroke (20.37%) and peripheral artery disease (4.76%). In overall population (n=800), bisoprolol (2.5 to 5mg/day) showed significant reduction in resting heart rate (RHR) (14bpm), and LVEF (5.08%). Similarly, in NOCAD cases significant changes in RHR (12.14bpm), and LVEF (4.68%) were noted at 24 weeks. Adverse events included chest congestion (6.61%), asthenia (5.03%), hypotension (4.76%), muscular weakness (3.70%), and bradycardia (1.85%) that were mild to moderate with none requiring treatment withdrawal.

Conclusion: Bisoprolol remains a clinically feasible option in Indian patients with NOCAD cases following percutaneous coronary intervention (PCI) as it reduces RHR and improves LVEF. Despite high rates of cardiovascular risk factors like age, type 2 diabetes and diffuse polyvascular disease, the drug was well-tolerated, with fewer adverse events. These results support the use of bisoprolol in managing NOCAD in Indian patients, highlighting its potential therapeutic uses to prevent further cardiac dysfunction.

## Introduction

Coronary artery disease (CAD) comprises a spectrum of clinical entities, emerging as the foremost cause of mortality and disability globally [1,2]. Notably, individuals of Indian descent exhibit the highest rates of CAD, with mortality linked to CAD in this population being 20-50% higher than in any other demographic [3]. According to the reports from the Global Burden of Diseases, Injuries, and Risk Factors Study on cardiovascular deaths, ischemic heart disease was identified as the primary cause of cardiovascular fatalities, while hypertensive heart disease was ranked as the fourth most significant contributor to cardiovascular mortality [1].

CAD is increasingly recognised as a significant factor contributing to the pathophysiology of type 2 myocardial infarction, which is characterized by a mismatch between oxygen supply and demand [4].

Based on the degree of obstruction or stenosis, CAD is categorized into obstructive CAD and nonobstructive CAD (NOCAD). Obstructive CAD is defined as any degree of stenosis exceeding 50% [5] in the left main coronary artery. In contrast, NOCAD refers to stenosis less than 50% [5,6] in the left main coronary artery of the heart. Approximately 50% of patients undergoing elective coronary angiography are found to have NOCAD. Patients with nonobstructive coronary arteries, exceeding 50%, often experience recurrent chest pain, diminished functional capacity, and a reduced quality of life, thereby increasing the risk of all-cause mortality and myocardial infarction (MI) [7-10].

The existence of NOCAD has traditionally been deemed "insignificant" in medical literature. However, this perception might be flawed, as earlier studies have highlighted that the majority of plaque ruptures leading to MI originate from non-obstructive plaques [11]. In recent decades, advancements in preventive and therapeutic approaches have markedly improved the prognosis for individuals affected by CAD and various cardiovascular conditions. Percutaneous coronary intervention (PCI) enhances survival, especially in patients with low to medium anatomical complexity of CAD and left main disease. Guideline-directed medical therapy, featuring β-blockers such as bisoprolol, is the preferred anti-anginal approach for managing coronary microvascular disease, a predominant factor in NOCAD [12,13]. Scientific evidence supports the sustained use of β-blockers, correlating with reduced mortality following PCI in acute MI patients [14].

According to 2021 American College of Cardiology (ACC) and American Heart Association (AHA) chest pain guidelines, the term CAD has been redefined to include coronary artery stenosis (irrespective of degree of stenosis) and ischemia with no obstructive coronary arteries (INOCA). INOCA is specifically characterized as patients exhibiting angiographic evidence of ischemia but lacking obstructive CAD during coronary angiography [5]. The occurrence of INOCA stems from a mismatch in myocardial oxygen supply-demand, attributed to underlying coronary microvascular dysfunction, coronary vasospasm, or a combination of both factors [15].

β-blockers exert their clinical effects by mitigating elevated resting heart rate (RHR), blood pressure, and reduced left ventricular ejection fraction (LVEF), which are well-established risk factors for CAD and indicative of increased mortality and morbidity [16,17]. Additionally, β‐blockers act by diminishing myocardial oxygen demand, enhancing the ischemic threshold, and ameliorating maladaptive left ventricular remodeling [15]. Elevated RHR adversely impacts the myocardial oxygen balance by elevating oxygen consumption and diminishing its supply [18,19]. Among the β-blockers, bisoprolol stands out as a selective β1 receptor antagonist with a well-established safety profile, exerting a dual effect in reducing elevated blood pressure and RHR, thereby presenting itself as a valuable option for preventing secondary cardiovascular events [19,20].

The current post-hoc analysis of the BISOCARD study was undertaken to acquire real-world evidence regarding the safety and effectiveness of bisoprolol in the management of patients diagnosed with NOCAD who underwent PCI.

## Materials and methods

Ethical consideration

The study was conducted in according to the principles originating from the Declaration of Helsinki (Brazil, October 2013), Good Clinical Practices for clinical research in India, 2005, International Conference of Harmonization Guideline for Good Clinical Practice E6 (R2), New Drugs and Clinical Trials Rules, 2019 and with Indian Council of Medical Research’s national ethical guidelines for biomedical and health research involving human participants, 2017. Before study initiation, ethics committee approval was sought from Suraksha Ethics Committee. The study was also registered with the Clinical Trials Registry of India (CTRI/2023/01/049287).

Study design and population

**Figure 1:**
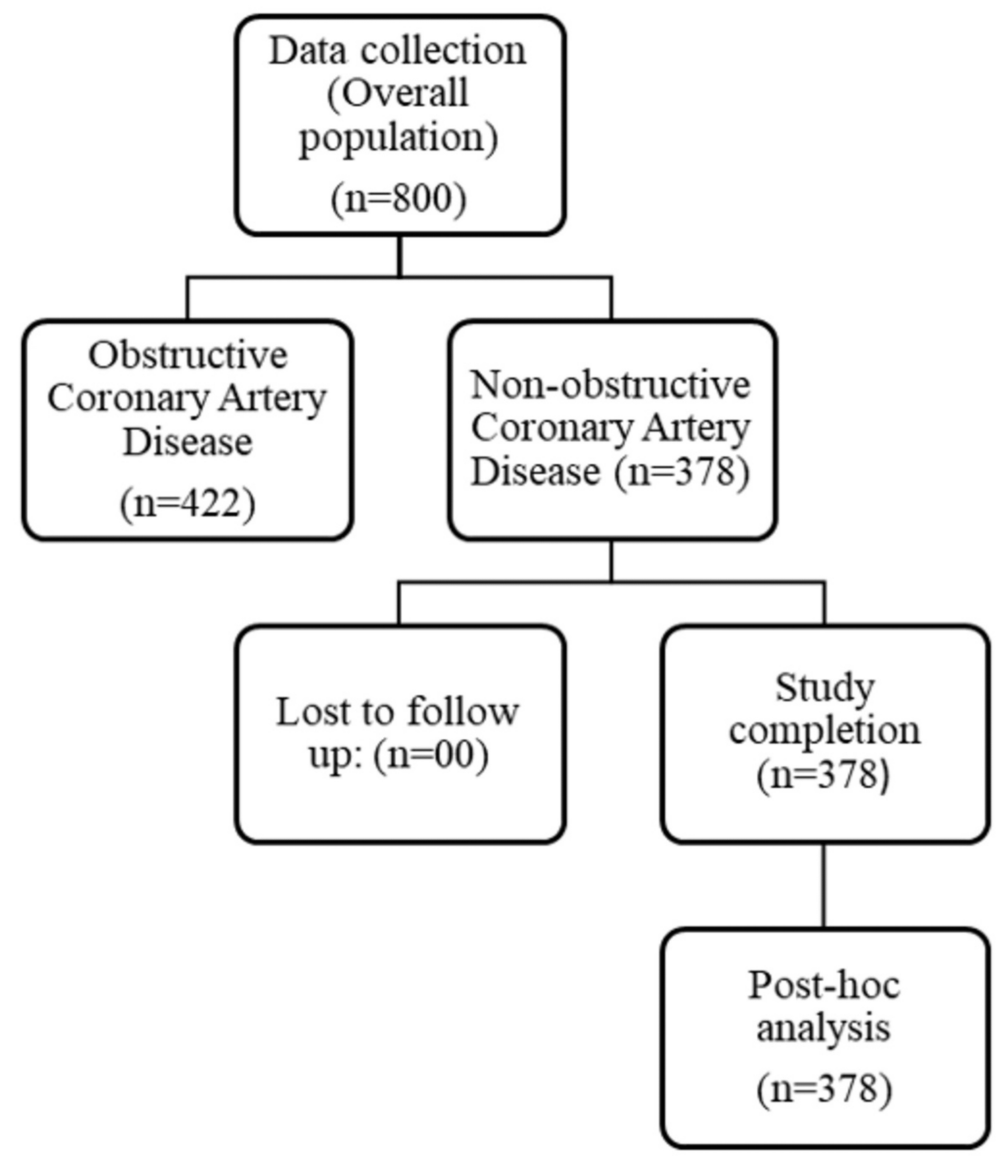
Patients' Disposition Chart

This was a post-approval, concurrent, observational, multi-centric, single-arm, open-label clinical investigation that enrolled 800 patients with CAD as intent-to-treat population (ITT) from 80 outpatient centres across India, offering a diverse patient pool. Specifically, per-protocol (PP) set of 378 (47.25%) cases of NOCAD were further analysed to gain real-world insights on safety and effectiveness of the study drug in this specific sub-group (Figure 1). Every participant enrolled in the study received a patient information sheet (PIS) which contained crucial study-specific details such as an explanation of the study's objectives, available treatment options, information on patient confidentiality and patient rights.

Inclusion criteria comprised both male and female patients aged 18 years or above, with known history of NOCAD along with comorbidities like hypertension and diabetes who required bisoprolol treatment, and had a 24-week follow-up at the same centre. Exclusion criteria encompassed patients who were hypersensitive to bisoprolol, those diagnosed with acute heart failure or needing intravenous inotropic therapy for decompensated heart failure episodes. Patients with cardiogenic shock, second- or third-degree atrio-ventricular block, sick sinus syndrome, or elevated hepatic enzymes (X3 upper limit of normal (ULN)) were also excluded. Additionally, pregnant, breastfeeding or sexually active women of childbearing age who did not practice approved birth control methods, were also not included in the study.

The primary objectives of the study include evaluation of change in mean values of RHR and LVEF after 24 weeks of treatment with the study drug bisoprolol. Secondary objectives include evaluation of change in mean values of systolic and diastolic blood pressure (SBP and DBP) after a 24-week treatment period and assessment for possible occurrence of treatment-emergent adverse events (TEAE).

Procedures

This real-world study was conducted between February 2023 and August 2023, with visits including baseline visit; the day of prescription of study drug, involved the explanation of the study's nature and risks to patients, along with assessment of inclusion and exclusion criteria, demographics, medical history, prior and concurrent medications, physical examination, and measurement of RHR, SBP and DBP and LVEF. Follow-up visit at 24 weeks marked the end of the study, involving the final assessment of RHR, LVEF, SBP, DBP and possible TEAE.

Given the real-life setting of the study, the study drug was prescribed by investigators, and patients acquired the drug from local pharmacies. Treatment compliance was monitored using case record forms, with patients considered to have completed the study if they adhered to the 24-week treatment period and underwent the follow-up assessments.

Intervention

The investigated drug was bisoprolol (CORBIS), available in strengths of 2.5mg, 5mg, and 10mg, manufactured by Torrent Pharmaceuticals Limited, Ahmedabad, India. Administration of the drug was intended orally, to be ingested whole with a full glass of water and without chewing. The frequency of tablet ingestion was determined by the treating physician (Table 1).

**Table 1:** Study Drug Details

Parameters	Treatment details
Study Drug	CORBIS 2.5 (Bisoprolol 2.5 mg) (Inactive Ingredients: Dibasic Calcium Phosphate, Microcrystalline Cellulose, Colloidal Silicon Dioxide, Pregelatinized Starch, Magnesium Stearate, Lake of Brilliant Blue, Titanium Dioxide, Triacetin, Hydroxy Propyl Methyl Cellulose, Ethyl Cellulose, Methanol and Methylene Chloride)
	CORBIS 5 (Bisoprolol 5 mg) (Inactive Ingredients: Dibasic Calcium Phosphate, Microcrystalline Cellulose, Colloidal Silicon Dioxide, Pregelatinized Starch, Magnesium Stearate, Yellow Oxide of Iron, Titanium Dioxide, Triacetin, Hydroxy Propyl Methyl Cellulose, Ethyl Cellulose, Methanol and Methylene Chloride)
	CORBIS 10 (Bisoprolol 10 mg) (Inactive Ingredients: Lactose Monohydrate, Microcrystalline Cellulose, Crospovidone, Magnesium Stearate, Hydroxy Propyl Methyl Cellulose, Polyethylene Glycol, Talc and Titanium Dioxide)
Dosage Form	Tablet
Dosage	As per direction of physician
Route of Administration	Oral
Manufacturer	Torrent Pharmaceuticals Ltd.

Statistical analysis

Statistical analyses were performed using SPSS version 29.0.1.0 (IBM Corp., Armonk, NY, USA) and GraphPad Prism (GraphPad Software, La Jolla, CA, USA). Descriptive statistics, including change from baseline was used to analyse continuous variables like RHR, DBP, SBP and LVEF and expressed as mean ± standard error (SE) values with 95% confidence interval. Frequency (n) and percentage (%) were reported for categorical variables like patient demographics and other baseline characteristics. Significance of continuous variables was assessed by employing Student’s paired t-test, using a two-tailed test and considering p-value <0.05 as statistically significant. Subjects who completed a 24-week evaluation period were considered in the analyses.

## Results

Demographics and baseline characteristics

In this present real-word assessment involving 800 patients (ITT) with CAD, 611 (76.37%) were males and 189 (23.63%) were females. Major risk factor in the population was co-existing hypertension in 100% cases. Three hundred seventy-eight patients with NOCAD (PP dataset) comprised 286 (75.66%) males and 92 (24.34%) females. None of the patients were lost to follow-up. Mean age for NOCAD cases was 58.63 years and weight was 75.49kg. Most prevalent risk factor included hypertension and dyslipidemia whereas the most prevalent comorbidity was type 2 diabetes mellitus (Table 2).

**Table 2:** Patient Demographics and Baseline Characteristics BMI, Body Mass Index; CAD, Coronary Artery Disease; n, Number of subjects; SE, Standard Error

Parameters		n=378 (Non-obstructive CAD)	n=800 (Overall population)
Demographics	Age (years) (mean, SE)	58.63 (0.52)	58.82 (0.35)
	Height (cm) (mean, SE)	165.19 (0.54)	165.32 (0.37)
	Weight (kg) (mean, SE)	75.49 (0.56)	75.38 (0.39)
	BMI (kg/m^2^) (mean, SE)	27.78 (0.22)	27.79 (0.17)
	Gender (n, %)	Male: 286 (75.66%)	Male: (n=611) (76.37%)
		Female: 92 (24.34%)	Female: (n=189) (23.63%)
Identified Risk Factors (%)	Hypertension	100%	100%
	Dyslipidaemia	51.85%	57.25%
	Sedentary lifestyle	40.21%	50.00%
	Alcoholism	31.75%	33.50%
	Smoking	24.07%	31.63%
	Obesity	17.46%	22.63%
Comorbidities (%)	Type 2 Diabetes Mellitus	59.52%	68.13%
	Stroke	20.37%	14.13%
	Congestive Heart Failure	11.64%	10.50%
	Peripheral Artery Disease	4.76%	6.13%
Concomitant Medications (%)	Anti-hypertensives	100%	100%
	Anti-diabetics	60.05%	70.88%
	Statins	41.00%	48.88%
	Anti-platelets	29.89%	38.50%
	Anti-obesity drugs	19.05%	17.75%
Duration of Coronary Heart Disease (n, %)	< 1 year	74 (19.58%)	169 (21.13%)
	1-3 years	153 (40.48%)	336 (42.00%)
	3-6 years	122 (32.28%)	215 (26.88%)
	> 6 years	29 (7.67%)	80 (10.00%)

Efficacy analyses

In the overall population (n=800), at 24 weeks treatment with bisoprolol showed significant mean changes in RHR (14.0 beats per minute (bpm)), SBP (17.39mmHg), DBP (9.88mmHg), and LVEF (5.08%) (Table 3).

**Table 3:** Improvement in RHR, SBP, DBP and LVEF in Overall CAD Patients at 24-Week Evaluation Period CAD, Coronary Artery Disease; CFB, Change from Baseline; DBP, Diastolic Blood Pressure; LVEF, Left Ventricular Ejection Fraction, RHR, Resting Heart Rate; SBP, Systolic Blood Pressure; SE, Standard Error Values at 24 weeks are significant at p<0.01 (derived using paired t-test).

Overall CAD patients (n=800)					
Parameters			Mean (SE)	p-value	95% Confidence Interval
RHR (bpm)	Visit 1	Baseline	88.20 (0.54)	<0.01	(-14.90, -13.10)
	Visit 2	24 Weeks	74.19 (0.44)		
	Individual CFB		-14.00 (0.46)		
	Individual % CFB		-14.00 (0.60)		
SBP (mmHg)	Visit 1	Baseline	146.57 (0.38)	<0.01	(-18.82, -15.95)
	Visit 2	24 Weeks	129.15 (0.34)		
	Individual CFB		-17.39 (0.27)		
	Individual %CFB		-10.09 (0.68)		
DBP (mmHg)	Visit 1	Baseline	92.05 (0.87)	<0.01	(-10.78, -8.98)
	Visit 2	24 Weeks	82.18 (0.45)		
	Individual CFB		-9.88 (0.73)		
	Individual %CFB		-9.14 (0.46)		
LVEF (%)	Visit 1	Baseline	46.67 (0.47)	<0.01	(4.55, 5.62)
	Visit 2	24 Weeks	51.76 (0.30)		
	Individual CFB		5.08 (0.46)		
	Individual %CFB		13.39 (0.53)		

Similarly, in 378 NOCAD cases, baseline measurements were recorded as: RHR at 88.3 bpm, SBP at 146.06 mmHg, DBP at 91.96 mmHg, and LVEF at 46.85%. Following a 24-week intervention, RHR reduced significantly by 12.14 bpm, SBP and DBP lowered by 16.30 mmHg and 9.05 mmHg respectively. Moreover, LVEF showed significant improvement by 4.68% from baseline (Table 4). At the 24-week evaluation, the population with a baseline LVEF ≥40% (n=295) demonstrated a 3.34% improvement, whereas the population with a baseline LVEF <40% (n=83) demonstrated a 9.43% improvement (Table 5). At 24 weeks greater improvement was observed in RHR in the overall cohort compared to NOCAD cases (Table 6).

**Table 4:** Improvement in RHR, SBP, DBP and LVEF in Non-obstructive CAD Patients at 24-Week Evaluation Period CAD, Coronary Artery Disease; CFB, Change from Baseline DBP, Diastolic Blood Pressure; LVEF, Left Ventricular Ejection Fraction, RHR, Resting Heart Rate; SBP, Systolic Blood Pressure; SE, Standard Error Values at 24 weeks are significant at p<0.01 (derived using paired t-test).

Non-obstructive CAD patients (n=378)					
Parameters			Mean (SE)	p-value	95% Confidence Interval
RHR (bpm)	Visit 1	Baseline	88.30 (0.92)	<0.01	(-13.45, -10.82)
	Visit 2	24 Weeks	76.15 (0.65)		
	Individual CFB		-12.14 (0.67)		
	Individual % CFB		-11.00 (0.96)		
SBP (mmHg)	Visit 1	Baseline	146.06 (1.23)	<0.01	(-18.11, -14.50)
	Visit 2	24 Weeks	129.67 (0.68)		
	Individual CFB		-16.30 (0.92)		
	Individual %CFB		-9.73 (0.58)		
DBP (mmHg)	Visit 1	Baseline	91.96 (0.62)	<0.01	(-10.15, -7.94)
	Visit 2	24 Weeks	82.92 (0.46)		
	Individual CFB		-9.05 (0.56)		
	Individual %CFB		-8.82 (0.63)		
LVEF (%)	Visit 1	Baseline	46.85 (0.55)	<0.01	(3.85, 5.51)
	Visit 2	24 Weeks	51.53 (0.51)		
	Individual CFB		4.68 (0.42)		
	Individual %CFB		12.56 (1.04)		

**Table 5:** Improvement in Baseline LVEF ≥40% and <40% in Non-obstructive CAD Patients at 24-Week Evaluation Period CAD, Coronary Artery Disease; CFB, Change from Baseline; DBP, Diastolic Blood Pressure; LVEF, Left Ventricular Ejection Fraction, RHR, Resting Heart Rate; SBP, Systolic Blood Pressure; SE, Standard Error Values at 24 weeks are significant at p<0.01 (derived using paired t-test).

LVEF ≥40% and <40%					
Parameters			Mean (SE)	p-value	95% Confidence Interval
Baseline LVEF ≥40% (n=295)	Visit 1	Baseline	50.82 (0.49)	<0.01	(2.42, 4.26)
	Visit 2	24 Weeks	54.17 (0.52)		
	Individual CFB		3.34 (0.47)		
	Individual % CFB		7.70 (0.97)		
Baseline LVEF <40% (n=83)	Visit 1	Baseline	32.75 (0.42)	<0.01	(7.90, 10.97)
	Visit 2	24 Weeks	42.18 (0.79)		
	Individual CFB		9.43 (0.77)		
	Individual %CFB		29.85 (2.45)		

**Table 6:** Correlation Between Overall Cohort and Non-obstructive CAD Patients CAD, Coronary Artery Disease; DBP, Diastolic Blood Pressure; LVEF, Left Ventricular Ejection Fraction, RHR, Resting Heart Rate; SBP, Systolic Blood Pressure % change comparison for RHR was significant at p=0.02 (derived using independent sample t-test for calculating p-value of overall cohort and non-obstructive CAD patients.)

Between Overall CAD Population and Non-obstructive CAD patients				
Parameters	RHR (bpm)	LVEF (%)	SBP (mmHg)	DBP (mmHg)
% change between Overall CAD patients and Non-obstructive CAD patients	13.31%	7.94%	6.24%	8.45%
p-value	0.02	0.42	0.35	0.25

Within the cohort of 378 patients, 214 (56.61%) patients presented with baseline SBP >140 mmHg. Their baseline parameters were as follows: RHR at 88.79 bpm, SBP at 162.37 mmHg, DBP at 97.49 mmHg, and LVEF at 46.57%. Following a 24-week therapeutic intervention, RHR significantly decreased to a mean of 76.59 bpm, SBP and DBP lowered to a mean of 136.66 mmHg and 85.70 mmHg respectively, while LVEF significantly improved to a mean of 53.06% (Table 7). The findings highlight the potential therapeutic benefits of bisoprolol in managing patients with NOCAD.

**Table 7:** Improvement in RHR, SBP, DBP and LVEF in Cases With Baseline SBP >140 at 24-Week Evaluation CAD, Coronary Artery Disease; DBP, Diastolic Blood Pressure; LVEF, Left Ventricular Ejection Fraction, RHR, Resting Heart Rate; SBP, Systolic Blood Pressure; SE, Standard Error Values at 24 weeks are significant at p<0.01 (derived using paired t-test).

Non-obstructive CAD with baseline SBP >140 mmHg (n=214)					
Parameters			Mean (SE)	p-value	95% Confidence Interval
RHR (bpm)	Visit 1	Baseline	88.79 (0.88)	<0.01	(-13.84, -10.53)
	Visit 2	24 Weeks	76.59 (0.63)		
	CFB		-12.18 (0.84)		
	% CFB		-12.00 (1.15)		
SBP (mmHg)	Visit 1	Baseline	162.37 (1.09)	<0.01	(-27.62, -23.63)
	Visit 2	24 Weeks	136.66 (0.73)		
	CFB		-25.62 (1.01)		
	%CFB		-15.32 (0.50)		
DBP (mmHg)	Visit 1	Baseline	97.49 (0.65)	<0.01	(-13.08, -10.57)
	Visit 2	24 Weeks	85.70 (0.51)		
	CFB		-11.83 (0.63)		
	%CFB		-11.54 (0.68)		
LVEF (%)	Visit 1	Baseline	46.57 (0.75)	<0.01	(5.44, 7.53)
	Visit 2	24 Weeks	53.06 (0.63)		
	CFB		6.49 (0.53		
	%CFB		17.05 (1.38)		

**Figure 2:**
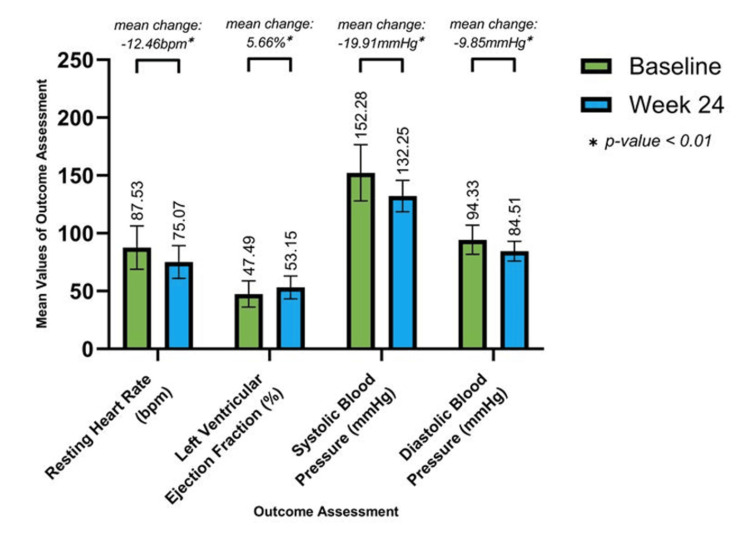
Improvement in Resting Heart Rate, Systolic Blood Pressure, Diastolic Blood Pressure and Left Ventricular Ejection Fraction in Cases With Prior History of Ischemic Events (n = 236) Values at 24 weeks are significant at p<0.01 (derived using paired t-test)

**Figure 3:**
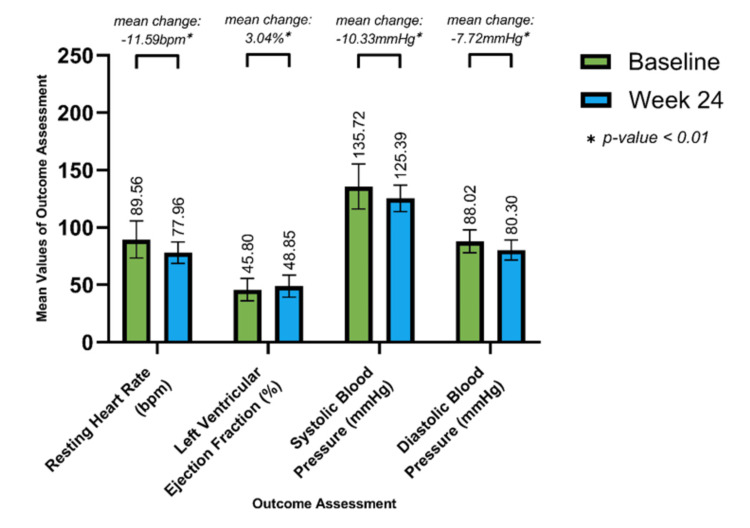
Improvement in Resting Heart Rate, Systolic Blood Pressure, Diastolic Blood Pressure and Left Ventricular Ejection Fraction in Cases Without Prior History of Ischemic Events (n = 142) Values at 24 weeks are significant at p<0.01 (using paired t-test)

Additionally, 236 (62.43%) patients in the NOCAD group presented with a prior history of ischemic events at baseline. After the 24-week treatment period, significant changes from baseline mean were observed in RHR (12.46bpm mean reduction), SBP (19.91mmHg mean reduction), DBP (9.85mmHg mean reduction), and LVEF (5.66% mean improvement) (Figure 2). Moreover, in 142 (37.57%) patients with no prior history of ischemic events, significant changes from baseline mean in RHR (11.59bpm mean reduction), SBP (10.33mmHg mean reduction), DBP (7.72mmHg mean reduction), and LVEF (3.04% mean improvement) were observed (Figure 3).

Safety analysis

Over a 24-week evaluation period, TEAEs were observed in 60 (15.87%) NOCAD individuals. These events included chest congestion (6.61%), asthenia (5.03%), hypotension (4.76%), muscular weakness (3.70%) and bradycardia (1.85%) that were mild and transient, requiring no treatment withdrawal.

## Discussion

The present study effectively justified its pre-determined objectives of reduction in crucial cardiac indicators including RHR, SBP, DBP, and LVEF following a 24-week treatment period with bisoprolol. Effectively managing NOCAD necessitates addressing underlying hypertension, elevated RHR, and reduced ejection fraction. β-blockers play a crucial role in enhancing endothelial functions, reducing RHR, thereby decreasing myocardial oxygen consumption and myocardial contractility, and increasing diastolic filling time. These pharmacological effects reduce the ischemic episodes and increase the threshold of ischemic symptoms [13,21,22].

A study encompassing 5713 previously healthy males without known or suspected heart disease unveiled that an RHR exceeding 75 bpm correlated with an elevated risk of sudden death nearly fourfold and all-cause mortality twofold [23]. The sub-group analysis of the BISO-CAD study, conducted by Chen YD et al., delved into the effects of bisoprolol on RHR in CAD patients with hypertension. It was evident that bisoprolol had a notable impact on reducing baseline RHR value after six, 12, and 18 months of treatment [19]. In the TENACITY study, which involved 400 patients with acute coronary syndrome, improvement in both RHR and LVEF was observed after treatment with bisoprolol. Specifically, the baseline RHR of 85.06 bpm decreased to 76.73 bpm, while the baseline LVEF of 41.45% increased to 48.73% following the treatment [24]. These findings are consistent with the present study, where bisoprolol significantly improved RHR, SBP, DBP and LVEF values.

In a study led by de Groote Pascal and colleagues, an investigation was undertaken on a consecutive series of patients characterized by LVEF below 40%. The study unveiled a substantial enhancement in LVEF, rising from an initial average of 31% to 41% (p<0.0001). This favourable outcome was associated with a reduction in ventricular volumes and an improvement in left ventricular filling function [25]. These findings closely align with the results of present study, where bisoprolol demonstrated its effectiveness in reducing LVEF over a 24-week treatment period. CIBIS-II (Cardiac Insufficiency Bisoprolol Trial II), a randomized controlled trial has demonstrated the clinical effectiveness of bisoprolol in reducing cardiovascular mortality and major adverse cardiovascular events among patients with left ventricular systolic dysfunction [26]. Çavuşoğlu Y et al. conducted a prospective, observational study where they concluded that RHR was significantly reduced in individuals who received β‐blocker therapy than those who did not (75.8 bpm vs. 80.4 bpm, respectively; p=0.001) [18]. A meta-analysis comprising of 72 studies compared the effects of β‐blockers with calcium channel blockers (CCBs) and found that β‐blocker usage was associated with fewer anginal episodes per week and lower rates of drug discontinuation, when compared to CCBs [27]. 

Safety outcomes from the meta-analyses have demonstrated that use of β-blockers has been associated with fewer adverse events compared to CCBs [28]. In the BISO-CAD study adverse events were observed in 163 patients, which accounts for 23.9% of the cases [19]. On the contrary in the present study a lower incidence of adverse events was reported, with only 60 cases (15.87%) of TEAEs.

The findings of the present study are in accordance with prior research studies conducted, reinforcing the rationale and benefits of incorporating bisoprolol treatment. Additionally, bisoprolol enhances crucial cardiac parameters, thereby, reducing cardiac workload and enhancing overall cardiac function.

The present post-hoc analysis has some limitations that should be acknowledged. Given the smaller sample size and the relatively short follow-up duration, there is a need for well-structured, appropriately designed controlled studies with long-term follow-up and diverse population. These studies could provide insights into the drug's safety and efficacy over prolonged periods of use, contributing to a deeper understanding of the clinical effectiveness and safety of the studied drug.

## Conclusions

In clinical practice, the secondary prevention treatment in patients with NOCAD varies largely. There is clearly need for trials investigating preventive treatments for NOCAD patients, and we conducted this post-hoc analysis of the BISOCARD study to meet this need. Bisoprolol demonstrated efficacy and well tolerability in preserving and improving cardiac function and parameters, including RHR, SBP, DBP, and LVEF in the Indian population with coexisting hypertension. These findings highlight an opportunity of adding bisoprolol to standard NOCAD treatment regimens to improve their quality of care.
